# Combination therapy with ampicillin and azithromycin in an experimental pneumococcal pneumonia is bactericidal and effective in down regulating inflammation in mice

**DOI:** 10.1186/1476-9255-11-5

**Published:** 2014-02-24

**Authors:** Arnab Majhi, Kiran Kundu, Rana Adhikary, Madhubanti Banerjee, Sayantika Mahanti, Anirban Basu, Biswadev Bishayi

**Affiliations:** 1 Department of Physiology, Immunology laboratory, University of Calcutta, University Colleges of Science and Technology, 92 APC Road, Calcutta 700009, West Bengal,India; 2 National Brain Research Centre, Manesar, Haryana 122051, India

**Keywords:** *Streptococcus pneumoniae*, Multiple drug resistance, Ampicillin, Azihromycin, Combination therapy, Inflammation

## Abstract

**Objectives:**

Emergence of multidrug resistance among *Streptococcus pneumoniae* (SP), has limited the available options used to treat infections caused by this organism. The objective of this study was to compare the role of monotherapy and combination therapy with ampicillin (AMP) and azithromycin (AZM) in eradicating bacterial burden and down regulating lung inflammation in a murine experimental pneumococcal infection model.

**Methods:**

Balb/C mice were infected with 10^6^ CFU of SP. Treatments with intravenous ampicillin (200 mg/kg) and azithromycin (50 mg/kg) either alone or in combination was initiated 18 h post infection, animals were sacrificed from 0 – 6 h after initiation of treatment. AMP and AZM were quantified in serum by microbiological assay. Levels of TNF-α, IFN-γ IL-6, and IL-10 in serum and in lungs, along with myeloperoxidase, inflammatory cell count in broncho alveolar lavage fluid, COX-2 and histopathological changes in lungs were estimated.

**Results:**

Combination therapy down regulated lung inflammation and accelerated bacterial clearance. This approach also significantly decreased TNF-α, IFN-γ, IL-6 and increased IL-10 level in serum and lungs along with decreased myeloperoxidase, pulmonary vascular permeability, inflammatory cell numbers and COX-2 levels in lungs.

**Conclusions:**

Combinatorial therapy resulted in comparable bactericidal activity against the multi-drug resistant isolate and may represent an alternative dosing strategy, which may help to alleviate problems with pneumococcal pneumonia.

## Background

*Streptococcus pneumoniae* (SP) is the most frequent isolate from clinical samples of respiratory tract infection, including acute exacerbation of chronic bronchitis and community acquired pneumonia (CAP) especially in the children and elderly [1,2]. Despite the availability of antimicrobial chemotherapy, the burden of pneumococcal disease in developing countries has changed very little over the last century [3]. The emergence and spread of penicillin resistant strains of SP has been reported from Europe, Asia, North America and Latin America [4,5] as well as in India [6]. Moreover an increasing trend in multidrug resistance (such as β-lactams, macrolides, doxycycline, and recently fluoroquinolone antibiotics) among these penicillin resistant SP has been a major concern worldwide for clinicians and has complicated the management of CAP [7,8]. Thus owing to antimicrobial resistance worldwide, national guidelines recommend a respiratory fluoroquinolone or combination antimicrobial therapy for outpatient treatment of CAP associated with risk factors for drug-resistant SP but the potential risks associated with these broad-spectrum regimens are justified with improved clinical outcome requires further study [9]. The usual first line antibiotics for bacterial respiratory infections are often macrolides (azithromycin, clarithromycin or erythromycin) in case of non-severe infections without risk factors for infection with drug-resistant pathogens, but in case of severe infections and chances of encountering multi-drug resistant SP, such monotherapy cannot be routinely recommended. Two of the most widely referenced guidelines for the management of CAP include those of the Infectious Disease Society of America (IDSA) and the American Thoracic Society (ATS) which recommends the use of a fluoroquinolone or a combination of β-lactam and macrolide for outpatients as well as for inpatients, non-ICU treatment [10-14]. Combination antibiotic therapy with different mechanism of action has been used to treat infections for decades with the goal of producing a wider spectrum, preventing the emergence of drug resistant subpopulations, reducing the dose of single agent, and achieving a synergistic effect. Retrospective studies of patients with bacteremic pneumonia have suggested that combination antibiotic therapy is associated with reduced mortality as compared with that seen among those who receive monotherapy [15-17]. In addition, most of the retrospective or observational studies regarding the use of a β-lactam and macrolide combination in treatment against pneumococcal bacteremia or CAP showed better outcome and lower mortality [18-23]. But data comparing the outcomes of the two most frequently recommended empirical antibiotic regimens for pneumococcal infection (combination antibiotic therapy with an extended spectrum β-lactam and a macrolide) for patients with severe CAP are sparse [24]. The efficacy and safety of intravenous azithromycin followed by the oral form, given in addition to intravenous ampicillin –sulbactam, evaluated in patients hospitalized due to CAP showed that this combination was effective and well tolerated [25]. It has been reported that an exposure to drugs such as beta-lactams, can cause rapid lysis of the Gram-positive bacteria, which leads to release of proinflammatory bacterial components and cytotoxins such as pneumolysins [26,27]. These are recognized by the innate immune system, triggering an inflammatory burst and potentially exacerbating the ongoing inflammation. In a model of pneumococcal secondary bacterial infection in mice, the β-lactam agent ampicillin was ineffective at reducing mortality despite rapid clearance of bacteria from the lungs [28], but treatment of mice with azithromycin reduced mortality. Moreover dual therapy with azithromycin and ampicillin against an azithromycin resistant strain was also able to cure secondary pneumonia in mice, which was independent of the antibacterial activity of azithromycin [29]. These studies suggested that there might be clinical benefit independent of antibiotic susceptibility pattern. We hypothesized that combination therapy with azithromycin and ampicillin against an isolate resistant to both the drugs might be able to exert its bactericidal and anti-inflammatory activities independent of antibiotic susceptibility pattern. Although results from *in vitro* studies are available [30], *in vivo* studies must be conducted to confirm the effectiveness of these combination therapy strategies against isolates resistant to commonly recommended regimens. SP isolates that were previously examined in human experimental colonization studies also colonized in inbred adult mice, thereby demonstrating the relevance of an animal model of pneumococcal pneumonia [31]. The aim of the present study was to test the *in vivo* efficacy of ampicillin and azithromycin alone and in combination against a multi-drug resistant strain of SP in an experimental murine pneumonia model. Now the question, whether a combination of ampicillin and azithromycin would be effective in treatment against an isolate of SP non-susceptible to penicillin and macrolide needs further investigation *in vivo*. Moreover, studying the experimental pharmacodynamic outcome associated with the combination therapy and evaluating the role of the physiological markers of inflammation like the level of proinflammatory (IL-6, TNF-α and IFN-γ) and anti-inflammatory cytokine IL-10 in the serum following combination antibiotic therapy, estimating the myeloperoxidase enzyme activity at the site of infection (as a marker of tissue neutrophil infiltration) and the level of expression of cyclooxygenase 2 (COX-2) in the lung tissue would also help to elucidate the mechanisms responsible for susceptibility to and pathophysiology of lung infection and regulation of those markers in inflammation.

## Methods

### Antimicrobial agents, media and bacterial strains

The study drugs which included ampicillin (AMP), azithromycin (AZM), amoxicillin/potassium clavulanate (AMC), oxacillin (OXA), ceftazidime (CAZ), cefotaxime (CTX), cefuroxime (CXM), ceftriaxone (CRO), clindamycin (CLI), imipenem (IPM), meropenem (MEM), levofloxacin (LVX), ciprofloxacin (CIP), rifampicin (RIF), vancomycin (VAN), trimethoprim/sulphamethoxazole (TMP-SXT), cefepime (FEP) and gentamicin (GEN) (HiMedia, Bombay, India), were used for all *in vitro* testing as per Clinical and Laboratory Standards Institute (CLSI) guidelines and the same AMP and AZM were used for intravenous injection in mice. The clinical isolate of *S. pneumoniae*, AMRI- SP-1, used for the experiment was obtained from the sputum of a patient with lower respiratory tract infection, admitted to Advanced Medicare and Research Institute (AMRI) hospital in Kolkata, West Bengal, India. A quality control strain of SP, ATCC 49619 was obtained as a kind gift from Dr. Indranil Roy, The Calcutta Medical Research Institute (CMRI), West Bengal, India. The strains were stored in skimmed milk tryptone glucose glycerol (STGG) medium (HiMedia, Bombay, India) at -80°C and subcultured twice onto Columbia blood agar plates (BAP) supplemented with 5% sheep blood (BioMe’rieux, Lyon, France) overnight at 37°C in 10% CO_2_ air incubator before use in all *in vitro* and *in vivo* experiments. All *in vitro* experiments were carried out in Mueller Hinton broth (MHB) (HiMedia, Bombay, India). Brain heart infusion broth (BHI) (HiMedia, Bombay, India) was used as the medium for pneumococcal cultures prior to experiments with mouse. All experimental samples were placed on Columbia BAP (BioMe’rieux, Lyon, France) supplemented with 5% sheep blood.

### In vitro susceptibility tests

Minimum inhibitory concentrations (MICs) and minimal bactericidal concentrations (MBCs) were determined by tube dilution method in MHB supplemented with 5% sheep blood. The tubes contained two fold dilutions of antibiotics and a final bacterial density of 10^5^ CFU/ml. The tubes were incubated for 18 h at 37°C. The MIC was defined as the lowest concentration of antibiotic at which no turbidity was visible to the naked eye. For determining MBC, 0.01 ml aliquots from tubes with no visible growth were plated onto BAP supplemented with 5% sheep blood and incubated overnight at 37°C. The MBC was defined as the lowest concentration of antibiotic that killed 99.9% of the original inoculums. Likewise disk agar diffusion test (DAD) was performed using Mueller Hinton agar supplemented with 5% sheep blood. The disk content of each drug, the number of antibiotics tested for MIC and MBC for the clinical isolate and for the quality control strain ATCC 49619 was done as per CLSI breakpoints for pneumonia. Modal values from three separate determinations were taken as the working values [32,33].

### Lung infection model

Male Balb/C mice (25 ± 2 g) were obtained from registered animal suppliers to the Department. Institutional Animal Ethical Committee (IAEC) reviewed and approved the methodology for use of these animals. All animals were maintained and utilized in accordance with recommendations from the IAEC and were provided with food and water *ad libitum*. After overnight incubation on BHI broth supplemented with 5% sheep blood, freshly grown colonies were suspended in fresh BHI broth supplemented with 10% filtered horse serum to an optical density of 0.12 at 550 nm. Experimental pneumonia was induced in the animals with a penicillin (MIC 64 μg/ml) and macrolide (MIC 8 μg/ml) resistant strain of *S. pneumoniae* AMRI-SP-1. Mice were anesthetized lightly by intravenous injection of ketamine hydrochloride (Sigma, Life Science) at 1 mg/kg of body weight through the tail vein, and 100 μl of a bacterial suspension (containing approximately 10^6^ colony forming units) was inoculated through the nares into the lungs of each mouse (50 μL per nostril). The advantage of intranasal inoculation is to mimic oropharyngeal aspiration, effectively infects upper and lower respiratory tract and is very simple. To investigate the change in colony forming units (CFU) in the lungs and blood, animals were sacrificed under ether anesthesia, from 18^th^ – 24^th^ h post infection. Blood was collected by cardiac puncture and their whole lungs were removed aseptically. The lungs were homogenized in 2 ml of sterile 0.9% saline, and the homogenates and blood were serially diluted 10-fold with sterile saline. 100 μL of the diluents of lung homogenates as well as blood was spread onto BAP supplemented with 5% sheep blood, and the plates were incubated at 37°C for 24 h. The numbers of CFU were determined by counting the numbers of single colonies that appeared on the plates showing alpha hemolysis (a characteristic specific to *S. pneumoniae*).

### Efficacy as assessed by bacterial density: Determination of bacterial loads in blood and lungs

Blood (0.5 ml) was obtained at 0 hours (immediately after administration of the drug), 1, 2, 3, 4, 5, and 6 hours (18-24 hours post infection) post antibiotic treatment after AMRI- SP-1 infection by cardiac puncture under ether anesthesia and exsanguinated at those selected intervals. The blood from each infected mice was diluted with sterile saline in 1:1 ratio and 100 μl of this diluted sample was plated on Columbia BAP supplemented with 5% sheep blood. At the previously mentioned time points post infection, bacterial loads in the lungs of SP infected mice were determined. For determination of the numbers of CFU in the lungs, lung tissues were dissected and homogenized in Hanks’ balanced salt solution without supplements by using a tissue homogenizer. The resulting homogenates of each sample were then plated in 10-fold serial dilutions on BAP, followed by incubation at 37°C for determination of the bacterial loads, as recently described in detail [34].

### Pharmacokinetic and pharmacodynamic studies

Pharmacokinetic (PK) and pharmacodynamic (PD) studies were conducted for AMP and AZM in mice. Concentration in sera was determined after administration through the tail vein a single intravenous dose of AMP at 200 mg/kg body weight and AZM at 50 mg/kg body weight. This dosage of ampicillin and azithromycin produces concentrations similar to those achieved in humans after an oral dose of 500 mg, showing concentrations in pulmonary tissues of mice that were above MIC for the organism for 48 to 72 h after injection. The drugs were administered through the tail vein in a volume of 100 μL per dose, 18 h after intranasal challenge with AMRI-SP1 [35]. At 0, 1, 2, 3, 4, 5 and 6 hours following a single dose of AMP or AZM or both in combination, blood samples were obtained from the mice in groups of three by cardiac puncture during ether anesthesia. After blood collection, samples were centrifuged at 5000 × g at 4°C and the serum was collected and stored at –80°C until it was analyzed. Antibiotic concentrations in serum were determined by the agar well diffusion method by using *Bacillus subtilis* ATCC 12432 as the bioassay reference strain. The zone diameter obtained were plotted against known antibiotic concentration comprising a suitable range on a semi-log graph paper to obtain a standard curve which was used to extrapolate the antibiotic concentration in serum samples at several time points as stated before. The antibiotic concentration in serum was then used to assess several PK and PD parameters. PK parameters assessed were C_max_ (μg/ml)_,_ defined as the peak plasma concentration of a drug after administration of a dose; C_min_ (μg/ml), defined as the lowest concentration that a drug reaches before the next dose is administered; area under the concentration curve (AUC)_0-6_, an integral of the concentration time curve (after a single dose or in steady state) measured in μg.ml^-1^.h^-1^; t_1/2,_ defined as the biological half-life, which is the time required for the concentration of the drug to reach half of its original value measured in hours; and k_e_, defined as the elimination rate constant which is the rate at which drugs are removed from the body measured in per hour. Among the PD parameters assessed were the AUC/MIC ratio, which takes both the antimicrobial concentration and time into account for predicting outcomes of concentration independent antibiotics, T > MIC, defined as the time period during which the serum antibiotic concentration remains above the MIC level measured in hours; C_max_/MIC is the ratio of maximum achievable concentration of the drug in serum to MIC.

### Protein binding in serum

We have assumed that unbound or free drug equilibrates with the extravascular space and that the total concentration of antibiotic in any given space is a combination of the free and protein bound drug has been considered for binding of protein in serum. Moreover the actual levels of free drug changes very little with alterations in binding to serum proteins of as much as 80% or 90%. Thus the total concentration of antibiotic in serum has been estimated for studying the in vivo efficacy of the therapy [36].

### Survival rate study

Determination of the efficacy of combination antibiotic therapy against pneumococcal pneumonia was first established in survival rate studies. Groups of 12 mice were inoculated intranasaly with *S. pneumoniae* as described above. Treatments with AMP at 200 mg/kg body weight and AZM at 50 mg/kg body weight either alone or in combination by intravenous route (through the tail vein) were initiated 18 hours post infection (p.i.). Control mice received sterile saline. Survival rate was recorded every 24 hour until day 3 p.i.

### Treatment regimens

18 hours after bacterial inoculation, groups of mice were treated with a single intravenous dose of either AMP (200 mg/kg body weight) or AZM (50 mg/kg body weight) only as monotherapy or administered both as combination therapy in a 0.1 mL volume, and sacrificed for sample collection at the previously stated time point, starting at 18th hours (0 h post antibiotic treatment) and continuing till 24th h (6 hours post antibiotic treatment) with an interval of 1 h in between two successive sampling point. Since the aim of the study was to see the bactericidal activity as will be determined by viable cell count and not the survival, the endpoint was chosen to be 6 hours after the initiation of therapy [37]. Mice receiving combination therapy received 0.1 mL of AMP, immediately followed by 0.1 mL of AZM. These dosing intervals were chosen so as to simulate the *in vivo* efficacy of short term high dose treatment of the drugs in humans. Untreated SP infected animals were considered as control and received same volume of isotonic saline (Additional file 1).

### MPO activity as a marker of neutrophil infiltration

Myeloperoxidase (MPO) enzyme activity was analyzed as index of neutrophil infiltration in the lung tissue, because it is closely related with the number of neutrophil present in the tissue. Blood free lung homogenates was homogenized and centrifuged at 3000 × g for 30 minutes at 4°C. MPO activity was estimated against a standard curve made with commercially available MPO, by methods previously described [38].

### Lung vascular permeability

The Evans blue permeability assay was used to quantify lung capillary permeability. Evans blue avidly binds to serum albumin and can therefore be used as a tracer for transcapillary flux of macromolecules. Evans blue (0.2 ml at a concentration of 25 mg/ml) was injected in a tail vein 30 min prior to the sacrifice. Lungs were homogenized in 2 ml of potassium phosphate buffer. Evans blue was extracted by incubating samples in 4 ml of formamide at 60°C for 24 h, followed by centrifugation at 5,000 × *g* for 30 min. The concentration of Evans blue was estimated by dual-wavelength (620 and 740 nm) spectrophotometry, which allowed correction of optical densities (E) for contaminating heme pigments. Thus, the following formula was used: E620 (corrected) = E620 - (1.426 × E740 + 0.03) [39].

### Cytokine levels in lung

For cytokine (IL-6, IL-10, IFN-γ and TNF-α) measurements, lung homogenates were lysed in lysis buffer pH 7.4 consisting of 300 mM NaCl/L, 15 mM TRIS/L, 2 mM MgCl2/L, 2 mM Triton X-100/L, 20 ng pepstatin A/mL, 20 ng leupeptin/mL, and 20 ng aprotinin/mL, and were centrifuged at 1500 × *g* for 15 min at 4°C; the supernatant was frozen at -20°C, until cytokine measurement by ELISA as per manufacturer’s protocol (Ray Biotech).

### Sample preparation for cytokine measurement from serum

Blood samples were transferred into micro-centrifuge tubes and allowed to clot at 4°C followed by centrifugation at 3000 × g for 5 min at 4°C. The supernatant pale yellow colored serum was pipette out carefully with the help of micropipettes into fresh micro centrifuge tubes, labeled and used for cytokine analysis. Serum from different groups were normalized to the protein content by Bradford method before the assay and levels of cytokines (IL-6, IL-10, IFN-γ and TNF-α) were determined by Sandwich ELISA according to the manufacturer’s instruction (Ray Biotech) in a Bio-Rad ELISA Reader.

### Expression of Cox-2 in lung tissue

Expression of cyclooxegenase-2 (cox-2) in lung tissues was determined by immunoblotting by methods described elsewhere [40].

### Inflammatory cells

Leukocyte recruitment to alveoli was determined in the broncho alveolar lavage fluid (BALF). Briefly, animals were sacrificed under ether anesthesia and trachea was exposed and intubated with a catheter, and then repeated 1 ml injections of PBS were made until a total of 3 ml of BALF was recovered. BALF was centrifuged at 3,400 × g for 10 min, and supernatant was frozen at -80°C until analysis of inflammatory mediators. Cells in the pellet were resuspended in PBS for quantification of leukocytes with a haemacytometer, and cell populations were enumerated from Diff-Quik Stain kit (Catalog No: NC9943455; Thermo Fisher Scientific Inc.) cytospin preparation [41].

### Histopathological examinations

Lung injury was observed by standard histological procedures [38]. Whole lungs were fixed in 4% formalin, embedded in paraffin, and processed for light microscopy using eosin and hematoxylin stainings.

### Statistical methods

The observers involved in data collection and analysis were not completely blind to treatment conditions. However, the methodology used for sample identification prevented subjective bias in the experiments. On the other hand, doses and animals were randomized to treatment conditions. Data was expressed as mean ± S.D. Means were compared between groups by using analysis of variance (ANOVA). P <0.05 was considered significant.

## Results

### Determination of MICs, MBCs and DAD for different antibiotics tested against S. pneumoniae

Median MIC values for different antibiotics against the isolate AMRI SP-1 and ATCC-49619 were determined in triplicate according to the CLSI micro dilution broth technique. The results obtained from MIC, MBC and DAD of the pneumococcal isolate and the reference strain are listed in Table 1.

**Table 1:** **In vitro susceptibilities of *Streptococcus pneumoniae *** strains to different antimicrobial agents*

**Drug**	**MIC (mg/L)**		**MBC (mg/L)**		**Zone diameter (mm)**	
	**ATCC 49619**	**AMRI SP 1**	**ATCC 49619**	**AMRI SP 1**	**ATCC 49619**	**AMRI SP 1**
PEN	0.06^a^	64^b^	0.06	-	26	+
AMP	0.25^a^	>32^b^	0.5	-	20	+
AZM	0.12^a^	8^b^	2	16	23	10
AMC	0.06^a^	2^a^	0.12	8	24	22
OXA	0.06^a^	0.25^a^	0.25	0.5	23	22
CAZ	8^c^	16^b^	16	>64	23	15
CRO	1^a^	2^b^	4	4	22	16
CTX	4^a^	0.5^a^	8	1	23	22
CXM	0.5^a^	2^a^	1	64	25	23
FEP	0.06^a^	0.5^a^	0.06	1	25	24
CLI	0.5^a^	4^b^	0.5	16	17	13
IPM	0.06^a^	0.12^a^	0.12	1	23	25
MEM	0.06^a^	0.06^a^	0.12	1	24	24
LVX	0.12^a^	16^b^	0.12	32	19	12
CIP	0.06^a^	1^a^	0.12	2	23	25
RIF	0.06^a^	0.12^a^	0.12	1	20	21
VAN	1^a^	>64^b^	4	-	19	+
GEN	0.5^a^	2	1	4	23	20
TMP-SXT	1^a^	>64^b^	2	-	18	+

*PEN- Penicillin, AMP- Ampicillin, AZM- Azithromycin, AMC- Amoxicillin/Potassium Clavulanate, OXA- Oxacillin, CAZ- Ceftazidime, CRO- Ceftriaxone, CTX- Cefotaxime, CXM- Cefuroxime, CLI- Clindamycin, IPM- Imipenem, MEM- Meropenem, LVX- Levofloxacin, CIP- Ciprofloxacin, RIF- Rifampin, VAN- Vancomycin, GEN- Gentamicin, TMP-SXT- Trimethoprim/Sulphamethoxazole.

‘-‘Not within the detectable limit; ‘+’ No zone of inhibition detected.

^a^Sensitive, b: Resistant, c: Intermediate.

### Murine pneumonia model

Administration of AMP in combination with AZM resulted in a significant reduction of colony-forming units in lungs from 2 to 6 hours, and in blood it was between 2- 4 hours post antibiotic treatment compared with non-treated infected animals. In addition, the lungs of mice treated concomitantly with AMP and AZM at 18 hours post infection had fewer *S. pneumoniae* organisms on 3, 4, 5 and 6 hours, respectively, after antibiotic treatment than those of mice treated with AMP or AZM alone (Table 2).

**Table 2:** **Bacterial burden in lungs and blood of mice infected with *S. pneumoniae***^***a ***^**and receiving either a single or a combined antibiotic treatment**

**Treatment**	**Collection of samples at different time (hours) after antibiotic administration**	**Total number of mice/number of bacteremic mice**	**CFU (Mean ± SD)**	
			**Lungs (per ml of tissue homogenate)**	**Blood (per ml)**
Untreated	0	4/0	114 ± 10.26	29 ± 9.34
	1	4/0	186 ± 12.04	38 ± 8.33
	2	4/1	290 ± 8.48	134 ± 10.06
	3	5/2	356 ± 15.23	182 ± 13.65
	4	6/3	408 ± 7.36	242 ± 15.27
	5	6/4	468 ± 11.45	286 ± 10.14
	6	6/6	480 ± 5.76	307 ± 10.58
AMP alone	0	4/0	390 ± 17.43	16 ± 5.66
	1	5/0	338 ± 15.27	24 ± 5.06
	2	5/0	307 ± 10.58	42 ± 8.19
	3	5/2	286 ± 10.14	186 ± 4.58
	4	5/3	242 ± 15.27	196 ± 8.50
	5	6/3	182 ± 13.65	143 ± 9.53
	6	6/4	134 ± 10.06	111 ± 3.05
AZM alone	0	4/0	376 ± 7.05	56 ± 6.28
	1	4/0	342 ± 11.59	71 ± 11.25
	2	4/0	311 ± 14.36	83 ± 10.09
	3	4/2	262 ± 9.53	162 ± 11.59
	4	5/3	196 ± 4.58	134 ± 6.02
	5	6/2	143 ± 8.50	104 ± 7.58
	6	6/2	111 ± 3.05	95 ± 3.51
AMP + AZM combined	0	4/0	381 ± 3.51	
	1	4/0	356 ± 9.60	89 ± 7.26
	2	4/1	324 ± 7.36	90 ± 12.03
	3	5/1	252 ± 4	175 ± 8.79
	4	6/1	173 ± 7.37	131 ± 6.82^*^
	5	6/0	137 ± 6.02	79 ± 5.36^*^
	6	6/0	99 ± 1.52	44 ± 10.15

^a^5 × 10^6^ CFU/mouse; ^c^Significant difference (P < 0.05) compared with the results for untreated *S. pneumoniae* infected group. Mean bacterial counts in lungs and blood at 18 h post infection for mice infected with *S. pneumonia* (AMRI-SP-1) and after initiation of antibiotic therapy either alone or in combination. ^*^Bacterial CFU counts in the AMP + AZM group are significantly lower than that of AMP or AZM alone groups at 4^th^ and 5^th^ hour post antibiotic treatment. Results for each group are given as mean ± standard deviation of five mice.

Table 2 also shows the changes in bacterial density in the lungs and blood of mice after infection with AMRI-SP1. Infected mice developed bacteremia within 24 hours of infection. The numbers of viable cells of AMRI-SP1 in the lungs and blood of untreated infected mice showed significant gradual increase in blood, up to 24 hours after infection, and their numbers also increased in lungs. Administration of AMP or AZM alone to infected animals significantly reduced bacterial counts in lungs and blood with time.

### Pharmacokinetics (PK) and pharmacodynamics (PD) of the drugs

Following a single intravenous bolus administration of AMP (200 mg/kg body weight) and AZM (50 mg/kg body weight), the PK and PD values obtained in the serum of mice infected with *S. pneumoniae* AMRI-SP1 is shown in Table 3.

**Table 3:** **Pharmacokinetic and pharmacodynamic parameters**^**a **^**for ampicillin (AMP) and azithromycin (AZM) following a single intravenously administered dose of both drugs 18 h post infection**

**Parameters**		
**(Mean ± SD)**	**Ampicillin**	**Azithromycin**
**Pharmacokinetics**		
Dose (mg/kg)	200	50
C_max_ (μg/ml)	4586 ± 22.3	8.9 ± 0.26
C_min_ (μg/ml)	16.25 ± 0.9	2.1 ± 0.18
AUC_0-6_ (μg.h/ml)	111265 ± 14610	15310 ± 1215.86
t_1/2_ (h)	0.963 ± 0.03	16.5 ± 1.56
K_e_ (h^-1^)	0.057 ± 0.013	0.19 ± 0.002
**Pharmacodynamics**		
AUC/MIC (h)	3477.03	32.24
T > MIC (h)	6.741	5.1
C_max_/MIC	143.31	1.11

^a^*Abbreviations*: *C*_max_ maximum concentration of drug in serum, C_min_ minimum concentration of the drug in serum, *t*_1/2_ time taken to reach half of the maximum concentration, AUC area under the concentration-time curve from time zero to 6 h post antibiotic treatment, *K*_*e*_- Elimination rate of the drug, *T*_*>MIC*_ Time during which the drug concentration remains above the MIC value in serum, *AUC/MIC* Ratio of area under the curve during 6 h of post antibiotic treatment to the MIC value for the S. pneumoniae strain AMRI-SP1,* C*_*max*_*/MIC* ratio of maximum concentration of the drug achieved in serum to MIC of the drug.

### Therapeutic efficacy of AMP and AZM combination against mortality in experimental pneumococcal pneumonia

**Figure 1:**
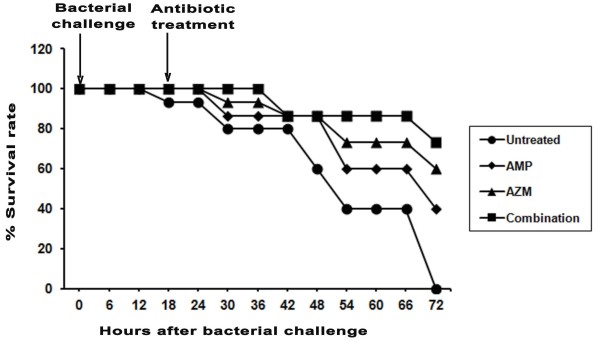
**Effect of combined antibiotic treatment on the survival of mice infected with *S. pneumoniae *(AMRI SP-1).** Mice were challenged with a low dose of *S. pneumoniae* (5×10^6^ CFU/mouse). Infected mice were treated with either AMP or AZM only or both in combination, 18 hours after infection (arrow: treatment regimen). The results (P < 0.05) of the survival analysis for groups of *S. pneumoniae* infected mice receiving either sterile saline (infected control) or antibiotic (n = 12 mice per experimental group and treatment regimen are indicated). AMP: Ampicillin treated; AZM: Azithromycin treated; Combination: AMP + AZM treated).

Inoculation of mice with 10^6^ CFU of *S. pneumonia* (AMRI SP-1) resulted in 100% mortality in untreated animals within 3 days post infection (Figure 1). AMP administered at 200 mg/kg body weight at 18 hours post infection was associated with ~40% survival rates where as therapy with AZM alone at 50 mg/kg body weight initiated at same time resulted in ~60% survival rate. Furthermore, treatment with both the antibiotics was associated with ~80 – 90% survival rates. (P < 0.05).

### Lung tissue myeloperoxidase (MPO) enzyme activity

**Figure 2:**
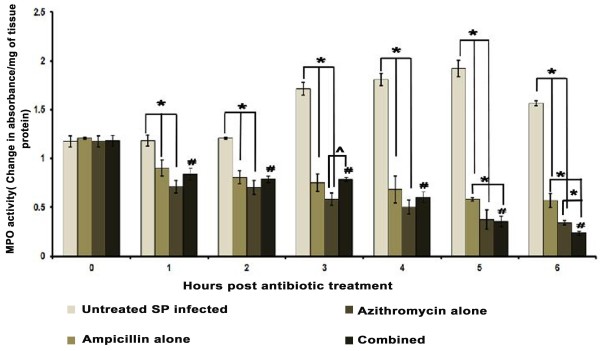
**MPO activity of lung tissue of mice after intranasal administration of *S. pneumoniae *(AMRI-SP 1) followed by treatment with ampicillin or azithromycin alone or in combination.** MPO activity was analyzed as index of neutrophil infiltration in the lung tissue. The rate of change in absorbance was measured spectrophotometrically at 405 nm. MPO activity has been defined as the concentration of enzyme degrading 1 μM of peroxide/min at 37°C and was expressed as change in absorbance/min. mg of protein. The results were reproduced in three repeated experiments. Data are expressed as mean ± SD of mice per group. P value less than 0.05 was considered as significant. *Significant decrease and ^#^Significant increase at P < 0.05 level.

The activity of MPO enzyme which is an indicator for neutrophil infiltration and the highest levels of lung MPO in infected animals appeared at 6 h. When AMP or AZM were administered alone or in combination, it caused significant (P < 0.05) time dependent reduction in tissue MPO enzyme activity than that of non-treated AMRI-SP1 infected mice (Figure 2).

### Pulmonary vascular permeability

**Figure 3:**
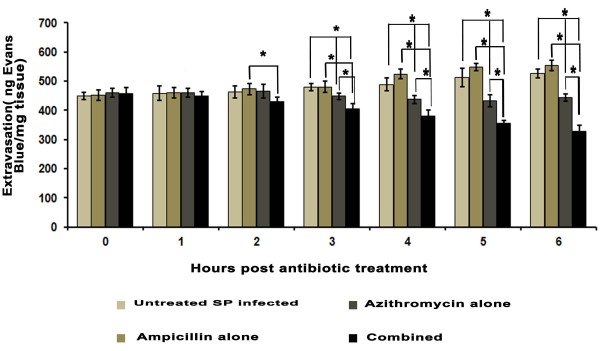
**Lung vascular permeability measurement.** Pulmonary vascular permeability in *S. pneumoniae* infected groups (mean ± SD for five mice). The results were reproduced in three repeated experiments. Data are expressed as mean ± SD of mice per group. P value less than 0.05 was considered as significant. *Significant decrease and ^#^Significant increase at P < 0.05 level. ^*^, SP infected vs. AZM alone; significant decrease at 3, 4, 5 and 6 hour, SP infected vs. AMP + AZM combined; significant decrease at 3,4,5 and 6 hour, SP infected + AZM vs. Sp infected + AMP + AZM; Significant decrease at 3,4,5 and 6 hour.

The pulmonary vascular permeability (as evaluated by Evans blue extravasations) showed higher values (P < 0.05) in *S. pneumoniae* infected untreated mice which was decreased gradually after treatment of AZM alone or in combination with AMP at 3,4,5 and 6 hours post antibiotic treatment (Figure 3).

### Cytokine (IL-6, IL-10, IFN-γ and TNF-α) levels in serum after treatment with combined antibiotics in AMRI-SP-1 induced experimental pneumonia

**Figure 4:**
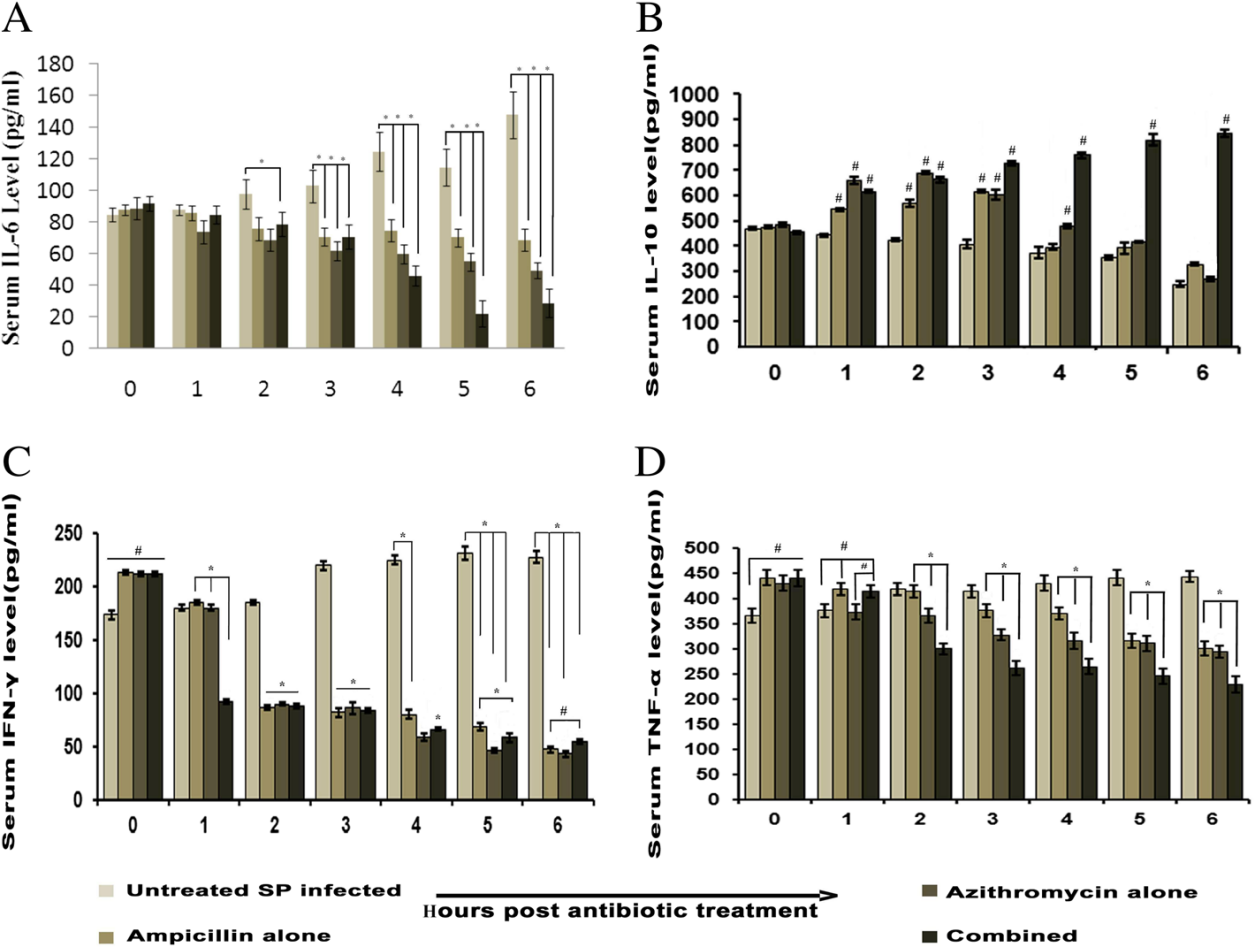
**Serum levels of IL-6 (A), IL-10 (B), IFN-γ (C), and TNF-α (D) in different groups of mice at 0 to 6 h post antibiotic treatment.** Levels of IL-6 **(A)**, IL-10 **(B)**, IFN-γ **(C)**, and TNF-α **(D)**, in serum from *S. pneumoniae* infected mice untreated or treated with ampicillin (AMP) or azithromycin (AZM) alone or in combination after 18 h post infection were determined by utilizing ELISA according to the manufacturer’s recommendations and were expressed from triplicate experiments. AMRI SP-1 infected animal which were left untreated was considered as control while comparing with those treated AMP or AZM or both. A significant increase in TNF-α, IFN-γ, and IL-6 but decrease in IL-10, ^*^P < 0.05 was observed after 1 h of post antibiotic treatment; *S. pneumoniae* isolate AMRI SP-1 alone, versus *S. pneumoniae* AMRI SP-1, + AMP, significant decrease in TNF - α, IFN-γ and increased IL-10, #P < 0.05, *S. pneumoniae* AMRI SP-1 alone, versus *S. pneumoniae* AMRI SP-1 + AMP + AZM showed significant decrease in TNF-α, IFN-γ and significant increase in IL-10 following 1 h post antibiotic treatment, #P < 0.05.

Serum TNF-α, IFN-γ, and IL-6 levels but not IL-10 was increased significantly after *S. pneumonia* infection (P < 0.05). Treatment of mice with either AMP or AZM alone or in combination after infection was able to significantly down regulate the serum TNF- α, IFN- γ and IL-6 levels at 2, 3, 4, 5 and 6 hours post antibiotic treatment. However, AMP in combination with AZM also increased the serum IL-10 level after 3, 4, 5 and 6 hour post initiation of therapy than that of AMP or AZM alone (Figure 4; A: IL-6; B: IL-10; C: IFN-γ and D: TNF-α).

### Cytokine (IL-6, IL-10, IFN-γ and TNF-α) levels in lung homogenates after treatment with combined antibiotics in AMRI-SP-1 induced pneumonia

**Figure 5:**
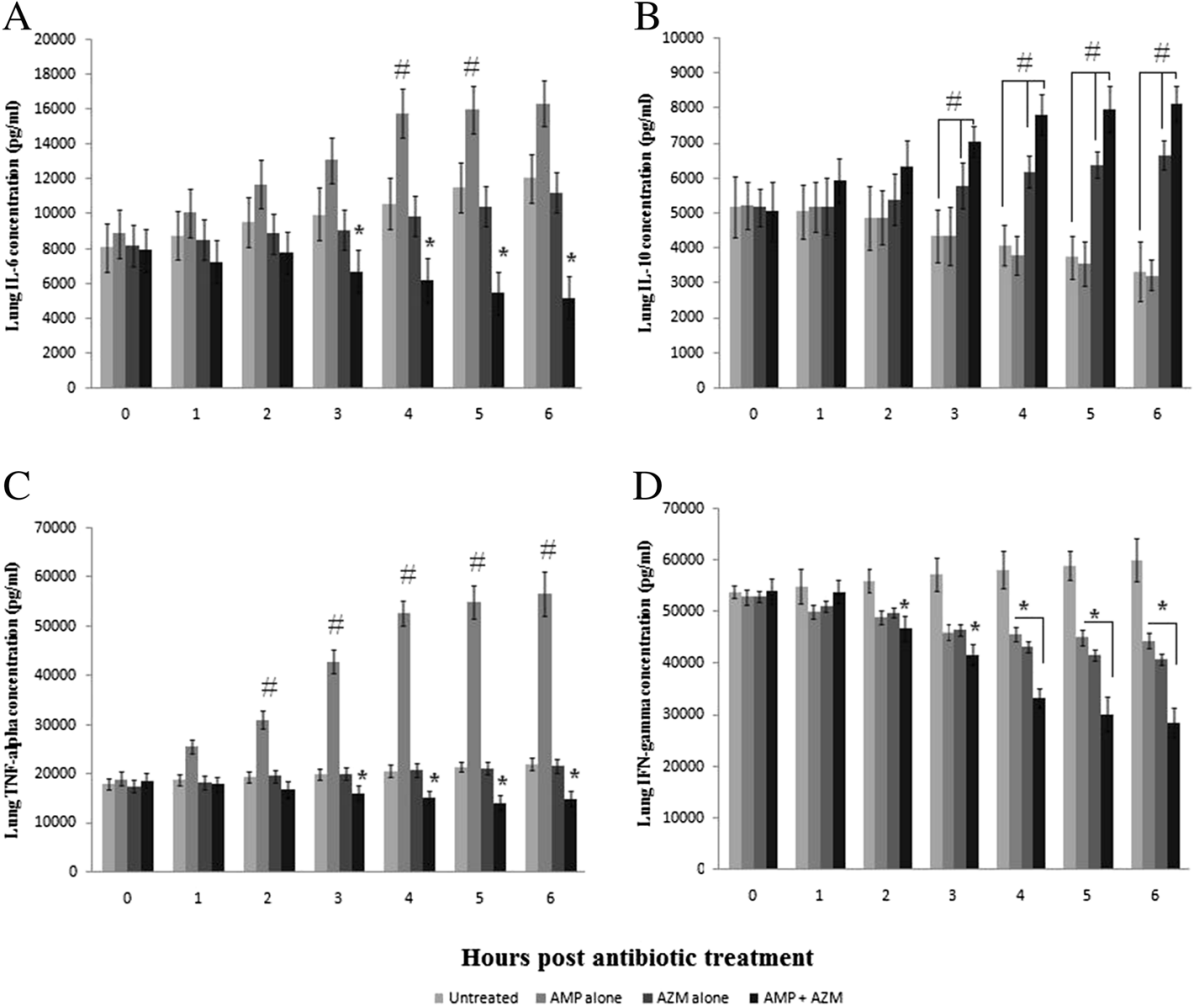
**Cytokine level in lung tissue.** Groups of mice (*n* = 21) were infected with AMRI-SP1 and were monitored for the development of pneumonia. 18 h post infection treatment with either AMP at 200 mg/kg or AZM at 50 mg/kg body weight, single dose of antibiotic treatment was initiated. Considering 18^th^ h to be the zero hour of antibiotic treatment, animals were sacrificed every hour continuing till 24^th^ h post infection (i.e. 6^th^ hour post antibiotic treatment). After administration of the single antibiotic dose, lungs were homogenized and assayed for estimation of cytokines. Levels of IL-6 **(A)**; IL-10 **(B)**; TNF-α **(C)** and IFN-γ **(D)** were determined and mean ± SD of values obtained were expressed in pg/ml from triplicate experiments. Untreated, *S. pneumoniae* infected; AMP, *S. pneumoniae* infected and treated with ampicillin; AZM, *S. pneumoniae* infected and treated with azithromycin; Combined, *S .pneumoniae* infected and treated with both ampicillin and azithromycin. ^#^, Significant increase or ^*^, Significant decrease in combined treatment group compared to monotherapy with AMP or AZM alone at P < 0.05.

As correlates of antibiotic treatment–mediated pulmonary inflammation, levels of cytokines in lung homogenates were measured. An increase in the levels of cytokines particularly TNF-α and IL-6 was seen in the lungs of AMP treated mice initiated 18 hours after *S pneumonia* infection, and was reduced after initiation of treatment with AZM alone or in combination with AMP. However, the lung IFN-γ was decreased at 2 hours after initiation of AMP or AZM alone or in combination, when compared to untreated *S. pneumonia* infected mice. Conversely, the level lung IL-10 were increased starting at 2 hours after initiation of AZM alone or in AMP plus AZM treated mice and sustained up to 6 hr post antibiotic treatment when compared to *S. pneumonia* infected untreated group (Figure 5; A: IL-6; B: IL-10; C: TNF-α and D: IFN-γ).

### Effect of AMP and AZM treatment on lung tissue Cyclooxygenase-2 level in the S. pneumoniae infected mice

**Figure 6:**
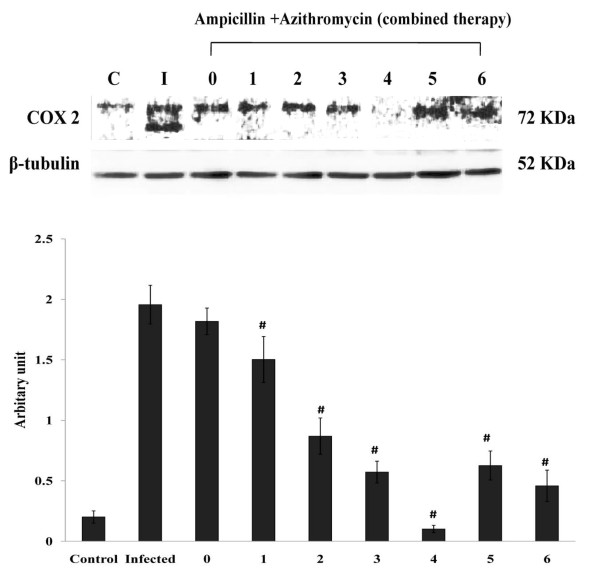
**Expression of COX-2 after treatment with ampicillin in combination with azithromycin in lung tissue.** Expression of COX-2 in lung tissue was measured in terms of fold change over *S. pneumoniae* infected untreated control. Highest level of COX-2 was found at 18 h post infection. Gradual reduction in COX-2 level was visible after treatment with ampicllin in combination with azithromycin with the decrease being most prominent at 4^th^ hour post antibiotic treatment. *S. pneumoniae* AMRI SP -1 infected untreated control group versus *S. pneumoniae* AMRI SP-1 + ampicillin + azithromycin treated group (P < 0.01 significant decrease with respect to SP infected untreated control at 1-3 h post antibiotic treatment).

Immunoblot analysis of lung tissue homogenate showed that COX-2 level was significantly increased at 18 hours post-infection in case of the *S. pneumonia* AMRI-SP-1, which was gradually decreased at 2-4th hrs of post antibiotic treatment. After treatment with ampicillin along with AZM, cox-2 level was decreased at 4th hour of antibiotic treatment (Figure 6).

### Estimation of inflammatory cells in BALF

**Figure 7:**
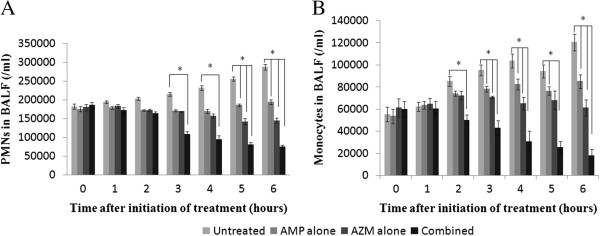
Mean (± SD) neutrophil (A) and monocytes (B) counts in BALF of infected mice, treated with AMP at 200 mg/kg and/or AZM at 50 mg/kg body weight. ^*^Significant decreases in both cell populations were seen by 3 hours in **(A)** and 2 hours in **(B)** after initiation of combination therapy, which was started after 18 hours post infection. *, P < 0.05.

Leukocyte recruitment to alveoli was determined in the BALF. Compared to *S. pneumoniae* infected untreated control group of mice that received antibiotic therapy either alone or in combination exhibited steady drop in PMN counts in BALF at every time point of the experiment. Furthermore combination therapy was more effective in down regulating PMN counts than monotherapy. A significant decrease in PMN recruitment occurred from 3 hours after initiation of therapy which corresponds to a gradual cure from bacterial invasion. As for the monocyte/macrophage recruitment in alveoli (BALF), a gradual increase was noted in untreated infected mice. A significant reduction in those cell counts was observed at 3 hours to 6 hours after initiation of treatment compared to either of the antibiotics alone (Figure 7).

### Lung histopathology

**Figure 8:**
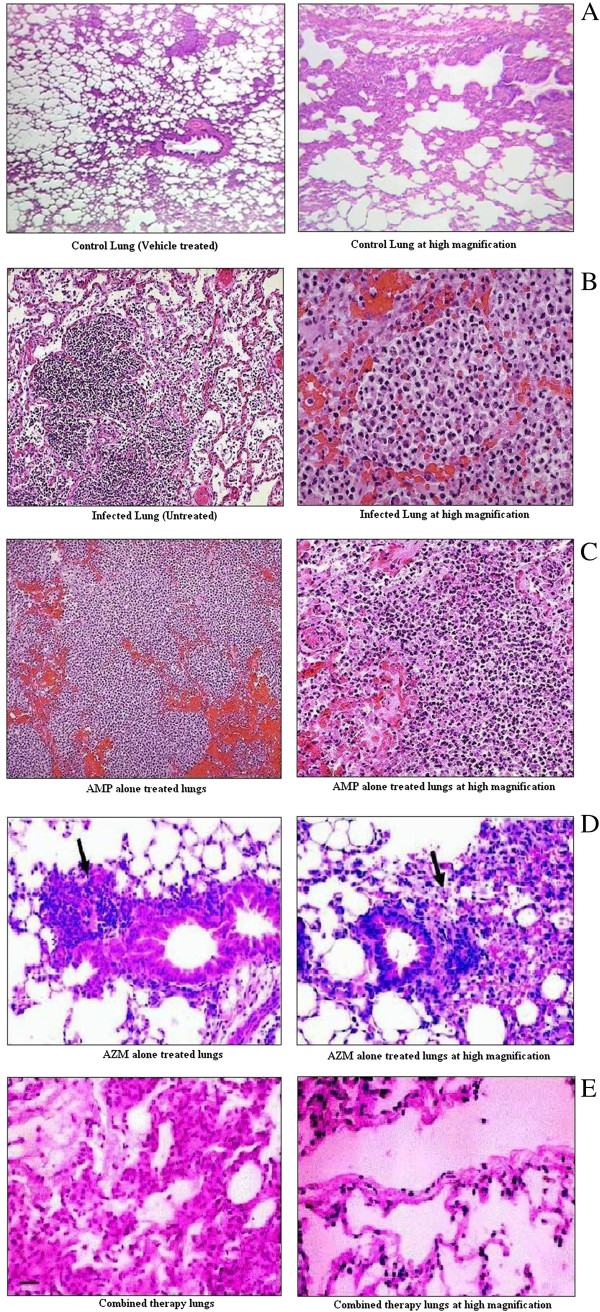
**Histology of lung tissue in normal mice (A); *S. pneumoniae *infected mice (B); treated with antibiotics ampicillin (AMP) (C) and/or azithromycin (AZM) (D) either alone or in combination (E) 18 hours post infection.** Pictures were taken from 0 – 6 hours after initiation of therapy which corresponds to 18 – 24 hours post infection. Profuse neutrophil count, diffuse edema with swelling of interstitium was noted in untreated infected animals, while mice receiving combination therapy recovered very fast than monotherapy and had tissue profiles similar to those of healthy controls. Left panel shows histology of lung tissue at low magnification and right panel at high magnification. Arrow indicates accumulation of PMNs in the alveoli.

To investigate the histopathological changes underlying *S. pneumoniae* induced experimental pneumonia in mice lungs and subsequent recovery from this disease state using combination therapy with AMP and AZM, animals were intranasally challenged with AMRI SP-1 and treated with antibiotics as mentioned before. Figure 8(A) shows normal lung histology of mice at low and high magnification. The sections of normal lungs shows alveoli are composed of a single layer of squamous epithelium, bronchioles are lined by ciliated columnar epithelium (larger bronchioles) or cuboidal epithelium (smaller bronchioles leading to alveoli). Between the alveoli a thin layer of connective tissue and numerous capillaries also lined with simple squamous epithelium.

Figure 8(B) shows lung histology of mice infected with AMRI SP-1 at 18 hours post infection at low and high magnification. At low magnification a patchy area of alveoli that are filled with inflammatory cells are seen. The alveolar structure is still maintained, which is why pneumonia often resolves with minimal residual destruction or damage to the lung. At high magnification the alveolar exudates of mainly neutrophils is seen. The surrounding alveolar walls have capillaries that are dilated and filled with RBCs.

Figure 8(C) shows lung histology as a result of treatment with AMP at low and high magnification. Destruction of lung tissue and haemorhage associated with the accumulation of more number of inflammatory cells are visible. At higher magnification, early abscessing pneumonia was observed. Alveolar walls are not clearly seen only sheets of neutrophils are visible.

Figure 8(D) shows histological changes in lungs of mice treated with AZM at low and high magnification. Representative lung histology demonstrates that AZM treatment led to persistent lung infection with extensive granulomas and peribronchiolar inflammation.

Figure 8(E) shows histological changes in lungs of mice treated with AMP and AZM in combination at low and high magnification. Animals treated with both the drugs recovered very fast and had tissue profiles similar to those of healthy controls. Since the combined drugs were administered once, few residual inflammatory cells were observed after therapy.

## Discussion

Approaches have been made to find new targets for antimicrobial activity, use of combination agents that are effective against more than one target in the cell, or new delivery mechanisms to maximize the concentration of antimicrobial agents at the site of infection, but relevant clinical evidence with respect to combining agents, has not been well elucidated for treatment against MDRSP strains. Given the astronomical costs involved in the research and development of a new drug and also the time required to take it from ‘the bench to the bedside’, utilization of combination therapy using known antibiotics should be a preferred as a cost-effective choice for therapy. In the current study we have used a murine pneumococcal pneumonia model to compare the efficacy of monotherapy with combination therapy by administering a single intravenous dose of AMP and AZM. From the bacterial growth and magnitude of inflammation (leukocyte infiltration into the lungs, lung cox-2 and high pulmonary vascular permeability) observed in our case supported the mouse model of pneumococcal pneumonia.

Use of β-lactam agents such as AMP, may increase and complicate the problem because these agents lyse the bacterial cell wall leading to release of proinflammatory substances such as cell wall components and cytotoxins which are recognized by the innate immune system and which trigger the inflammatory response [42,43]. It was observed that macrolides (erythromycin) and macrolide-like agents (AZM, clindamycin, telithromycin), at sub-MIC concentrations, were potent inhibitors of pneumolysin production by both susceptible and resistant strains of *Streptococcus pneumoniae*, with doxycycline being somewhat less effective, while amoxicillin, ceftriaxone, and tobramycin were ineffective. AZM alone is unlikely to be preferred as resistance rates of community isolates of *S. pneumoniae* are high [44]. But owing to its anti-inflammatory effects and broader spectrum of activity it might be a realistic candidate [45-48]. In addition AZM retained its anti-inflammatory activity against a resistant strain when used in combination therapy. This finding suggests that there might be clinical benefit independent of antibiotic susceptibility pattern [29].

Azithromycin (AZM) and ampicillin (AMP) in combination against an azithromycin resistant strain was reported to cure secondary pneumonia in mice. Thus we choose AZM and AMP as combinatorial antibiotic therapy even though we found the *S. pneumoniae* (AMRI-SP-1) was resistant to AMP or AZM applied in single doses. Furthermore, in a murine model of secondary, influenza-associated pneumococcal pneumonia, the lowest survival rate in antibiotic-treated animals was observed in those treated with AMP only, while the highest rates were noted in those treated with inhibitors of protein synthesis (AZM or clindamycin) only, or in combination with AMP [49]. Improved survival with AZM was associated with an attenuated inflammatory response, manifested as lower numbers of inflammatory cells and pro-inflammatory cytokines in the lungs, and less severe histopathological changes. Therefore, antibiotic selection based solely on the grounds of antimicrobial potency may be inappropriate in some clinical settings, particularly serious infections caused by toxin-producing pathogens with high bacterial loads [50]. In this situation, circumstances permitting, administration of an inhibitor of bacterial protein synthesis, either prior to, or together with a compatible bactericidal agent may be justified to reduce the potential risk of an antibiotic-associated inflammatory reaction. Based on laboratory, experimental animal, and limited clinical data, potential strategies to address this complex clinical problem include combining an inhibitor of bacterial protein synthesis (preferably one with secondary anti-inflammatory properties, *i.e.* a macrolide), with a cell-wall active agent. Thus, our choice of AMP along with AZM as combinatorial therapy against the multi-drug resistant *S. pneumoniae* (AMRI-SP-1) in this mouse model of pulmonary infection was hypothesized to be an effective combination therapy. AZM exhibits anti-inflammatory activities independent of its antimicrobial properties [51]. This antibiotic resulted in clinical cure in *S. pneumonia* infected mice, although it is unclear whether the improved outcomes are solely the result of the mechanism of action or whether they are the result of this factor in addition to the anti-inflammatory properties of the drug [49]. The exact mechanisms of action for the macrolides like azithromycin that have this anti-inflammatory action are still not completely defined, although it is known that they act by various molecular, cellular, and bacterial mechanisms. It might be due to decreased chemotaxis, migration, and cellular activity in neutrophils and macrophages and concomitant decrease in IL-6, TNF-α, IFN-γ and PGE2 in the air way passages after azithromycin administration.

Determining the drug levels in serum as a function of time is essential for estimating the concentration of the antibiotic that are necessary to inhibit (MIC) or to be bactericidal (MBC) to microorganisms. Drug concentration in the blood (plasma, serum) has been correlated to *in vivo* bacterial eradication. β- lactam antibiotics such as AMP are unevenly distributed in tissue, with a tissue: serum ratio < 1:1 for most sites. They are distributed mostly in the blood and extracellular fluid that represents about 20% of the total body mass. Conversely macrolides have high tissue: serum ratios (> 2:1) and are found predominantly inside cells. Concentrations of these drugs are therefore lower extracellularly while concentrations of β-lactams are higher [52]. AMP has been known to exhibit-time dependent killing which means a long time above MIC (T > MIC) or a large ratio of area under the curve (AUC) to MIC (AUC/MIC) is predictive of a successful treatment outcome [53]. Concentration dependent drugs like AZM are characterized by a steeper pharmacodynamic (PD) function; the steeper the PD function, the more efficient is the bacterial killing which increases commensurately with antibiotic concentration.

The PK and PD parameters suggest that ampicillin was widely distributed in the extracellular fluid and into tissues. A rapid distribution of the drug between blood and the extravascular tissue compartment was achieved which was consistent with that found in the literature. Azithromycin remained in circulation for a longer duration and was available in the tissue bed or at the site of infection thus exerting its bactericidal and anti-inflammatory effect there. It was reported that amoxicillin (AMX), a β-lactam antibiotic, was able to clear the infection of two resistant pneumococci (MICs 1 and 2 μg/ml) if the dose was increased [53]. However, in a mouse pneumonia model, significant bactericidal effect was not achieved on penicillin resistant pneumococci strains for which the MIC was ≥ 2 mg/L, even with a dose/MIC ratio of 200 [50]. In another study with penicillin resistant pneumococci strain (MIC 4 mg/L), a killing of 2 to 3 log_10_ within the first 6 h was observed, independent of C_max_ ranging from 2 to 20 times the MIC. Regrowth occurred after 12 h in a majority of the experiments [54]. Thus an increased C_max_ and larger AUC were not sufficient to achieve a predictable killing for that strain. The findings from our present study also supports this observation that AMP though administered at a 4 times greater dose compared to AZM, achieved a greater C_max_ and AUC but was not effective in clearing the bacterial load from the lungs in group of mice treated with AMP alone. So the need for studying highly resistant pneumococci is paramount to seek an explanation for this observation and determine its prevalence.

Macrolides induce a biphasic effect on the host. First, they have direct antimicrobial activity by stimulating the host defense against bacteria via stimulation of leukocyte degranulation, phagocytosis and oxidative burst. Secondly, after the acute infection, neutrophils that are primed by cytokines or pneumolysin are inhibited by macrolides, that leads to amelioration of the inflammatory response. Another potential explanation for the beneficial effects of macrolides is reduction in bacterial load with less cell wall lysis than beta-lactam antibiotics; this results in a more gradual reduction in bacterial load and, therefore, a more gradual release of immunologically reactive components, which may prevent an extended systemic inflammatory response [55]. In our study, changes in bacterial density in lung for AMP or AZM monotherapy were similar against *S. pneumoniae* isolate. For the AMP + AZM combination regimens, we found changes in lung bacterial density to be greater compared with AMP or AZM monotherapy for AMRI-SP-1. Clearance of bacteria from blood also increased after combined antibiotic therapy than the AMP or AZM alone at 3 h after initiation of antibiotic therapy. Therefore, it may be suggested that the outcome in pneumococcal pneumonia was improved when combination antibiotic therapy was initiated at early stages of *S. pneumoniae* infection even when the bacteria was resistant to that antibiotic [16,17].

Subsequently, we determined whether neutrophils were critical for combined antibiotic-mediated protection in the pneumococcal infection. Animals infected with *S. pneumoniae* and 24 h later that were remained untreated showed heavy infiltration of PMN affecting the lung inflammation. However, co-administration of AMP and AZM after the pneumococcal challenge led to reduced PMN infiltration in lungs.

The timing of the observed decrease in neutrophil numbers and inflammatory mediators argues against a causal link between decreased inflammation and host protection. Specifically, combined antibiotic treatment reduced the pulmonary bacterial burden as early as at 2 h after initiation of antibiotic treatment, whereas the decreased inflammatory response was also apparent until 6 h post antibiotic treatment. In addition to this discrepancy in timing, there is substantial evidence that the recruitment of neutrophils and regulatory release of proinflammatory mediators are protective against *S. pneumoniae-*induced mortality.

High IL-6 concentrations were found in the lungs of mice infected with SP. In addition relatively higher serum IL-6 levels has been reported after intravenous injection of wild type SP than after administration of pneumolysin (PLY) negative mutant SP suggesting the induction of an inflammatory response in the pulmonary compartment in the early phase of pneumococcal pneumonia [56]. During lung inflammation in acute phase, damaged alveolar capillary and epithelial membranes by PMN leading to leakage of protein rich edema fluid into the alveolar space, and formation of hyaline membranes which impaired gas exchange have been reported [57]. At early stages of infection the permeability of lung vasculature is increased due to enhanced release of proinflammatory cytokines (TNF-α, IFN-γ and IL-6) [58-60]. Hence, decrease in extravasations after initiation of combined antibiotic therapy after 3 h of post antibiotic treatment may be due to reduced lung TNF-α, IFN-γ and IL-6 level and increased anti-inflammatory cytokine (IL-10), which is sustained until 6 hours post antibiotic treatment.

The inflammatory cytokine response in the lung is characterized by intense elevation IL-6, TNF-α and IFN-γ which was decreased after combined treatment. A subsequent increase in IL-10 after combinatorial treatment, which is an anti-inflammatory cytokine that inhibits macrophage and neutrophil production, is the beginning of the anti-inflammatory response that prevents an uncontrolled inflammatory response. IL-6 has been considered as a marker for the severity of bacterial challenge represents a relevant marker for the evolution of a host response and high IL-6 concentrations have been found in the lungs of mice infected with SP [61]. Therefore, reduced IL-6 in combined antibiotic treated mice might be responsible for decreased inflammation in mouse lungs along with reduced lung TNF-α and IFN-γ after antibiotic treatment.

We observed that IFN-γ, TNF-α, IL-6 but not IL-10 production was increased initially 18 hours post-infection and decreased gradually thereafter following treatments with AMP and AZM. Therefore, it is likely that increased TNF-α and IFN-γ released into the circulation after infection by the administration of *S. pneumonia* cells or their exotoxins demonstrated a detrimental effect on the host. We found that severity of pneumonia is associated with altered balance of inflammatory cytokines, and conversely, altering the balance of inflammatory cytokines has a significant impact on the severity of pneumococcal pneumonia. It was reported that azithromycin at concentrations of 1, 5 and 10 μg/ml have been demonstrated to affect in various degree of production of IL-1, IL-6 and IL-10, GMCSF and TNF-α by human monocytes. Most remarkably, azithromycin resulted in a significant decrease of TNF-α in 100% of individuals and treatment with clarithromycin resulted in a significant decrease in IL-6 and TNF-α in 86% of individuals respectively [62,63].

Of several pneumococcal pneumonia-related molecular pathways with anti-inflammatory actions, we chose to focus on IL-10 as a representative of cytokine in this class. IL-10 appears to be valuable for attenuating inflammatory damage to human lung [64]. Since serum cytokines were considered as a reflection of inflammation induced by pathogens anti-inflammatory cytokines like IL- 10 continues to increase even at 6 hours after treatment of mice with AMP and AZM. This IL-10 level increment dictates the resolution of inflammation and may be a positive prognostic indicator for recovery of pneumonia due to the combined therapy. IL-10 inhibits the production of reactive oxygen and reactive nitrogen intermediates when monocyte and macrophages are activated by IFN-γ and therefore may be important in determining the outcome of pneumonia. As lack of IL-10 causes impaired clearance of bacteria leading to a more destructive cause of pneumonia, therefore, this elevated IL-10 in the combined antibiotic treated mice might be essential for efficient elimination of bacteria and therapy for protection against pneumococcal pneumonia. IL-10 is often considered as the master regulator in immunity from infection [65]. IL-10 reduces both the extent and the duration of inflammation, the outgrowth of pneumococci, and mortality [66].

Therefore, the present finding indicated that in the presence of concurrent treatment with AMP + AZM may lead to elevated circulating IL-10 that might influence bacterial outgrowth, suggesting that only in the latter phases of pneumococcal pneumonia is IL-10 essential for host defense. It was reported that IL-10 given at latter stages of infection prevented severe inflammation and lung edema and facilitated bacterial clearance in mice treated with ceftriaxone [66]. However, whether elevated systemic IL-10 during combined therapy could modulate the blood and lung levels of antibiotics [67], either AMP or AZM have not been tested in our case. Previous data also indicated a beneficial role for IL-10 as an adjunctive therapy to antibiotics against pneumococcal pneumonia in mouse model [66]. These protective effects might have resulted from decreased pulmonary inflammation and better availability of the drug to the infected sites. Better bacterial clearance was also reported in other in vivo studies with IL-10 [68].

Immunoblot analysis of lung tissue homogenate showed that COX-2 level was significantly increased at 18 h post-infection in case of the *S. pneumonia* (AMRI-SP1), which was gradually decreased at 1, 2, 3 and 4 h post antibiotic treatment. After treatment with AMP along with azithromycin, COX-2 level was significantly decreased on 4 h post therapy. Similar reduction in prostaglandin, nitric oxide, TNF-α, and IL-6 levels has been previously reported in murine macrophages treated with 5 to 80 μM of azithromycin [46]. Given its constitutively expressed nature and predominant role in prostaglandin synthesis during bacterial infection, potential strategies for drug resistant bacteria based on COX pathways or inhibiting COX-2 [69]. These data collectively support that combinatorial antibiotic treatment mediated COX-2 inhibition or strategies that disrupt prostaglandin signaling pathways as useful adjunctive therapies in treating persistent and multi-drug resistant infection.

The combined antibiotic therapy promoted the infiltration of peripherally circulating neutrophils into the lungs, leading to bacterial clearance, COX-2 pathway in lungs and the lung cytokines might determine the outcome of interactions with microbes in the lungs. Insights into the functional roles of cytokines, and regulatory factors in mediating pulmonary immune responses may contribute to rationally designing and appropriately using therapeutic and prophylactic agents. This basic study may be considered for strategies aimed at altering leukocyte recruitment, bacterial clearance and pulmonary inflammation in order to improve host defense (e.g. against antibiotic-resistant organisms).

## Conclusion

These data indicated a beneficial role for AMP and AZM as combinatorial therapy against pneumococcal pneumonia. Inflammation mediated by bacterial toxins on lysis of the cells due to exposure to cell wall active agents might be reduced with this mode of therapy in penicillin and macrolide resistant isolates also as evident from our findings, irrespective of their antimicrobial susceptibility pattern in *in vitro* conditions. Thus macrolides specifically azithromycin can be still used in combination with cell-wall active agents such as ampicillin in treatment of *S. pneumoniae* infections due to a resistant organism.

## Abbreviations

SP: *Streptococcus pneumoniae*; MDR: Multiple drug resistance; CAP: Community acquired pneumonia; AMP: Ampicillin; AZM: Azithromycin; TNF-α: Tumor necrosis factor-alpha; IFN-γ: Interferon gamma; IL: Interleukin; COX-2: Cyclooxegenase-2; BAP: Blood agar plate; BHI: Brain heart infusion; MIC: Minimum inhibitory concentration; MBC: Minimal bactericidal concentration; DAD: Disk agar diffusion; ATCC: American type culture collection; CFU: Colony forming units; PK: Pharmacokinetic; PD: Pharmacodynamics; AUC: Area under the concentration; PMN: Polymorphonuclear leukocytes; BALF: Broncho alveolar lavage fluid.

## Competing interests

For the manuscript entitled “Combination therapy with ampicillin and azithromycin in an experimental pneumococcal pneumonia is bactericidal and effective in down regulating inflammation in mice” by Arnab Majhi et al authors declared that they have no conflict of interest for this manuscript towards submission in *Journal of Inflammation.* The authors also state that we do not have a direct financial relation with the commercial identities mentioned in this manuscript that might lead to a conflict of interest for any of the authors.

## Authors’ contributions

Authors AB and BB designed the study and designed protocol. AM, RA, MB and SM performed all the experiments. KK performed the immunoblot for COX-2. AM, RA and MB managed the literature searches and analyses. AM undertook the statistical analysis; BB and AM wrote the manuscript. All authors contributed to and have approved the final manuscript.

## Supplementary Material

Additional file 1 Animal grouping and experimental time schedule. Click here for file
