# Supplementary figures and images for: Combination therapy with ampicillin and azithromycin in an experimental pneumococcal pneumonia is bactericidal and effective in down regulating inflammation in mice
Source: J Inflamm (Lond). 2014 Feb 24;11:5. doi: 10.1186/1476-9255-11-5 (PMC3936873; doi:10.1186/1476-9255-11-5)

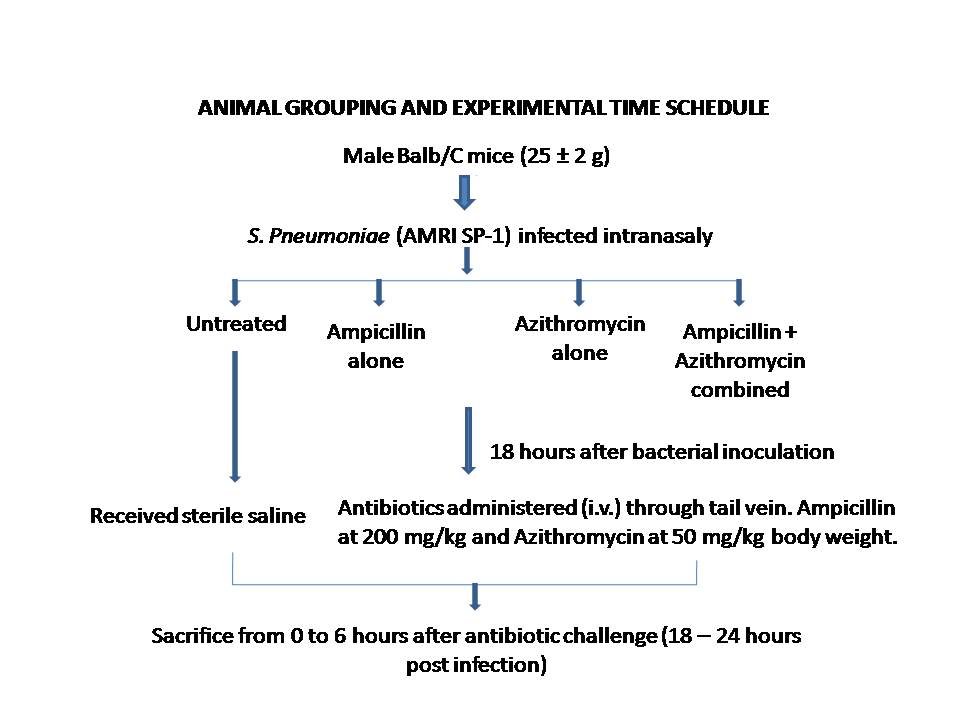

Supplement: Additional file 1 — Animal grouping and experimental time schedule. [file 1476-9255-11-5-S1.jpeg]
